# SEM characterization and ageing analysis on two generation of invisible aligners

**DOI:** 10.1186/s12903-021-01676-z

**Published:** 2021-06-23

**Authors:** Roberta Condò, Gianluca Mampieri, Aldo Giancotti, Loredana Cerroni, Guido Pasquantonio, Andrea Divizia, Annalisa Convertino, Barbara Mecheri, Luca Maiolo

**Affiliations:** 1 Department of Clinical Sciences and Translational Medicine, University of Rome “Tor Vergata”, Viale Oxford 81, 00133 Rome, Italy; 2 PhD in Nursing Sciences and Public Health, University of Rome “Tor Vergata”, Via Montpellier, 1, 00133 Rome, Italy; 3 Institute for Microelectronics and Microsystems - National Research Council, Unit of Rome, Via del Fosso del Cavaliere, 100, 00133 Rome, Italy; 4 Department of Chemical Science and Technologies, University of Rome “Tor Vergata”, Via della Ricerca Scientifica, 1, 000133 Rome, Italy

**Keywords:** Invisalign® aligner, Polymer, Scanning electron microscopy, Ageing tests

## Abstract

**Background:**

The purpose of the in vitro study is to investigate and compare the morphological features and the chemical stability in weight of two different polyurethane-based blends, Smart Track (LD30) and Exceed30 (EX30), used for orthodontic aligners manufacture before and after the oral usage.

**Methods:**

Twenty orthodontic aligners were randomly selected: 10 LD30 and 10 EX30, each group was divided in two subgroups, never used and intra-orally aged. By the employment of a Stereomicroscope, a section of 5 × 5 mm was cut from the buccal surface of the incisal region of each aligner. All samples were subjected to Scanning Electron Microscopy and Ageing tests in different solutions to simulate the hostility of the oral environment. The statistical method used was t-test.

**Results:**

At SEM images, LD30 appears more homogeneous in texture respect to EX30. However, after clinical usage, both materials show significant structural alterations: findings have been supported by higher magnifications at SEM, by which it is clearly to observe many superficial cracks cross through the polymer structures of LD30U, absent in never used samples. LD30U surface becomes also smoother due to the disappearance of most of the conglomerates, but at the same time also rougher while EX30U shows a greater irregularity and porosity in which large and deep cracks are also highlighted. Although these changes occur persistently, in the aging tests no significant weight loss from both materials has been found, confirming the initial hypothesis of a good chemical stability and safety of both polyurethane mixtures even in conditions of severe hostility.

**Conclusion:**

LD30 is the expression of the technological evolution of EX30, this is made evident above all by its morphological architecture, more homogeneous and defined but also by the chemical stability that can be appreciated even in evident critic situations.

## Background

In the modern manufacturing technology of orthodontic appliances, thermoplastic polymers are widely used as main materials due to their advantageous chemical, optical and mechanical properties, such as biocompatibility, translucency and transparency, high elasticity and good deformability, in addition to their great versatility, simplicity in processing, superior formability and low production cost [1–3].

Polymeric materials owe their evolution to the development of modern synthetic chemistry in the eighteenth century, so they are considered relatively recent materials. The mixing of most thermoplastic polymers can be used as an ordinary method to obtain a single product with more advantageous features. To date, a significant amount of literature has been published on various mixtures of thermoplastic materials, such as polycarbonate and polypropylene, rather that polyurethane and polycarbonate blends [4, 5].

Mixing polymers is an effective method of improving the mechanical properties of the polymers themselves: polyester, polyurethane and polypropylene are precisely the dominant materials in the polymer blends used for the production of transparent orthodontic aligners [6]. Thermoplastic materials ranking, lists polymers in amorphous, crystalline and liquid crystalline. Amorphous polymer possesses irregularly arranged molecular structure. Generally, in these materials molecular packing results very poor. Conversely, some polymers exhibit a regularly arranged molecular structure. In these materials, the chemical structure allows the polymer chains to fold on themselves and pack together in an organized manner. The resulting organized regions show the behavior characteristics of crystal. By a structural point of view, the crystalline domains act as a reinforcing grid, like in a composite material, thus improving the performance of the polymer over a wide range of temperatures. These are known as semicrystalline polymers since they maintain in their structure amorphous regions. In the different applications, it is essential to understand what is the perfect balance between these regions [7].

*Exceed30 (EX30),* was the polymer material used to realize Invisalign® aligners from 2001 to the beginning of 2013 [8–10]. It was an implantable medical grade polymer, made of polyurethane methylene diphenyldiisocyanate 1,6-hexanediol, tested for safety and biocompatibility in accordance with the United States Pharmacopeia, Class IV, Size 0.03" (0.75 mm) [11].

From the first quarter of 2013, EX30 has been replaced by a new innovative polymer called Smart Track (LD30), a multilayer aromatic thermoplastic polyurethane/co-polyester.

In a previous study a common constituent polyurethane-based constituent, and an additional element that justifies the difference in its mechanical properties, have been observed in LD30 composition. In particular, LD30 exhibits a more amorphous structure and a greater elastic recovery than EX30, revealing also a smaller residual deformation. The crystallinity reduction of LD30 respect to EX30 explains its higher translucency even after the clinical use [12]. In never used aligners, aesthetic features and transparency seem to remain the same compared to the previous material EX30 but given its better clinical performance, LD30 has become the standard Invisalign® material for all the various treatment options [13, 14]. It shows higher flexibility providing a gentle and more constant force reducing in pain intensity, duration and pressure upon aligner insertion and a more long-term clinical action. It also presents a better adaptability and adhesion to dental arch improving comfort and easiness in use [15]. Therefore, the change in the material used in the production phase of these orthodontic aligners seems to be strongly motivated by the careful search for a new polymeric blend that is shown to be improved in chemical-physical performance.

In light of these more recent findings, the null hypothesis of the present in vitro study has assumed that the morphological features and structural stability in LD30 are improved also, compared to those observable in EX30. Therefore, Scanning Electron Microscopy (SEM) analysis and accelerated aging tests are performed with the aim to qualitatively and quantitatively evaluate materials changes in order to understand the effective time in which the aligners lose their clinical efficiency, while ensuring their biological safety.

## Methods

Twenty Invisalign® aligners were selected for experimental tests. They included 10 reference aligners collected before intraoral placement and 10 taken from aligners worn intra-orally for 2 weeks, approximately 22 h a day. More in detail, all samples consisted of 10 LD30 aligners, including 5 never used (LD30N) and 5 used (LD30U), collecting from five different LD30 treatment sets of aligners, and 10 EX30 aligners, including 5 never used (EX30N) and 5 used (EX30U) deriving from five diverse EX30 treatment sets of aligners. By the employment of a stereomicroscope, a section of 5 × 5 mm was cut from the buccal surface of the incisal region of each aligner, obtaining a total of 28 samples double-blind selected. The different samples have been selected adopting the statistical analysis reported in tab.3 obtaining an average of 25 mm^2^ and an error of 0.25 mm^2^.

### SEM characterization

To obtain a deeply investigation of their morphology, specimens were previously prepared according to the following protocol. Each sample was treated with acetone to eliminate possible impurities, dehydrated and subjected to the process of metallization preceded by fastening on stubs. In this phase, the samples have been covered with a thin film of gold (Sputtering process) for 4 min with Bio-Rad SEM coating system Microscience Division. Once all the samples have cooled down, they were preserved in glass containers. The images were acquired by a Leo Supra 35 FE-SEM field emission Scanning Electron Microscope (Carl Zeiss, Germany).

### Ageing analysis

To investigate ageing effects of the two polymeric blends, the weight of used and never used samples have been measured to verify the chemical stability of the aligners. For this purpose, two different solutions, simulating the aggressive ambient of the oral cavity, have been prepared: Phosphate Buffered Saline (PBS) and 10 M Orthophosphoric acid [H_3_PO_4_] (Table 1).

**Table 1:** Solutions simulating the aggressive ambient of the oral cavity

Testing solutions	Description
PBS	Composed of potassium chloride [KCl], potassium phosphate monobasic [KH_2_PO_4_], sodium chloride [NaCl] and sodium phosphate dibasic [Na_2_HPO_4_]
Orthophosphoric acid	Molar concentration of 10 M [H_3_PO_4_]

New aligners carried out from the original package and used ones, wore by patients, have been prepared for EX30 and LD30 materials. Thus the experiment was organized with six samples of LD30N and LD30U and six of EX30 material new and used (EX30N, EX30U). Each sample has been immersed in PBS and H_3_PO_4_ at different time intervals: 3, 5 and 12 h (Table 2).

**Table 2:** List of ageing tests in PBS and H_3_PO_4_

Samples	Solutions	Ageing time (h)
LD30N	PBS	3, 5, 12
	H_3_PO_4_	3, 5, 12
LD30U	PBS	3, 5, 12
	H_3_PO_4_	3, 5, 12
EX30N	PBS	3, 5, 12
	H_3_PO	3, 5, 12
EX30U	PBS	3, 5, 12
	H_3_PO_4_	3, 5, 12

In particular, every sample has been placed into an Eppendorf test tube, immersed in its corresponding ageing solution at fixed temperature of 37 °C, at the three established time intervals. Different samples for each material have been tested and each sample has been weighted 5 times with a precision balance (Mettler Toledo) after each time interval. In order to compare samples of different size and weights a percentage weight relative variation has been calculated. The average and the standard deviation of the relative percentage weight variation have been presented (Table 3).

**Table 3:** Relative error

*Relative error*	
For LD30x	$\varepsilon ^{{t_{x} }} _{{STXrel}} = \frac{{LD30X_{{t_{x} }} - \mu (LD30X_{{t_{0} }} )}}{{\mu (LD30X_{{t_{0} }} )}}$
For EX30x	$\varepsilon ^{{t_{x} }} _{{EXXrel}} = \frac{{EX30X_{{t_{x} }} - \mu (EX30X_{{t_{0} }} )}}{{\mu (EX30X_{{t_{0} }} )}}$
*Mean values*	
For LD30x	$\mu (\varepsilon ^{{t_{1} }} _{{LD30Xrel}} )$; $\mu (\varepsilon ^{{t_{2} }} _{{LD30Xrel}} )$;$\mu (\varepsilon ^{{t_{3} }} _{{LD30Xrel}} )$
For EX30x	$\mu (\varepsilon ^{{t_{1} }} _{{EX30Xrel}} )$; $\mu (\varepsilon ^{{t_{2} }} _{{EX30Xrel}} )$;$\mu (\varepsilon ^{{t_{3} }} _{{EX30Xrel}} )$
*Standard deviation*	
For LD30x	$\sigma (\varepsilon ^{{t_{1} }} _{{LD30Xrel}} )$; $\sigma (\varepsilon ^{{t_{2} }} _{{LD30Xrel}} )$;$\sigma (\varepsilon ^{{t_{3} }} _{{LD30Xrel}} )$
For EX30x	$\sigma (\varepsilon ^{{t_{1} }} _{{EX30Xrel}} )$; $\sigma (\varepsilon ^{{t_{2} }} _{{EX30Xrel}} )$;$\sigma (\varepsilon ^{{t_{3} }} _{{EX30Xrel}} )$

## Results

### Scanning electron microscopy results

**Fig. 1:**
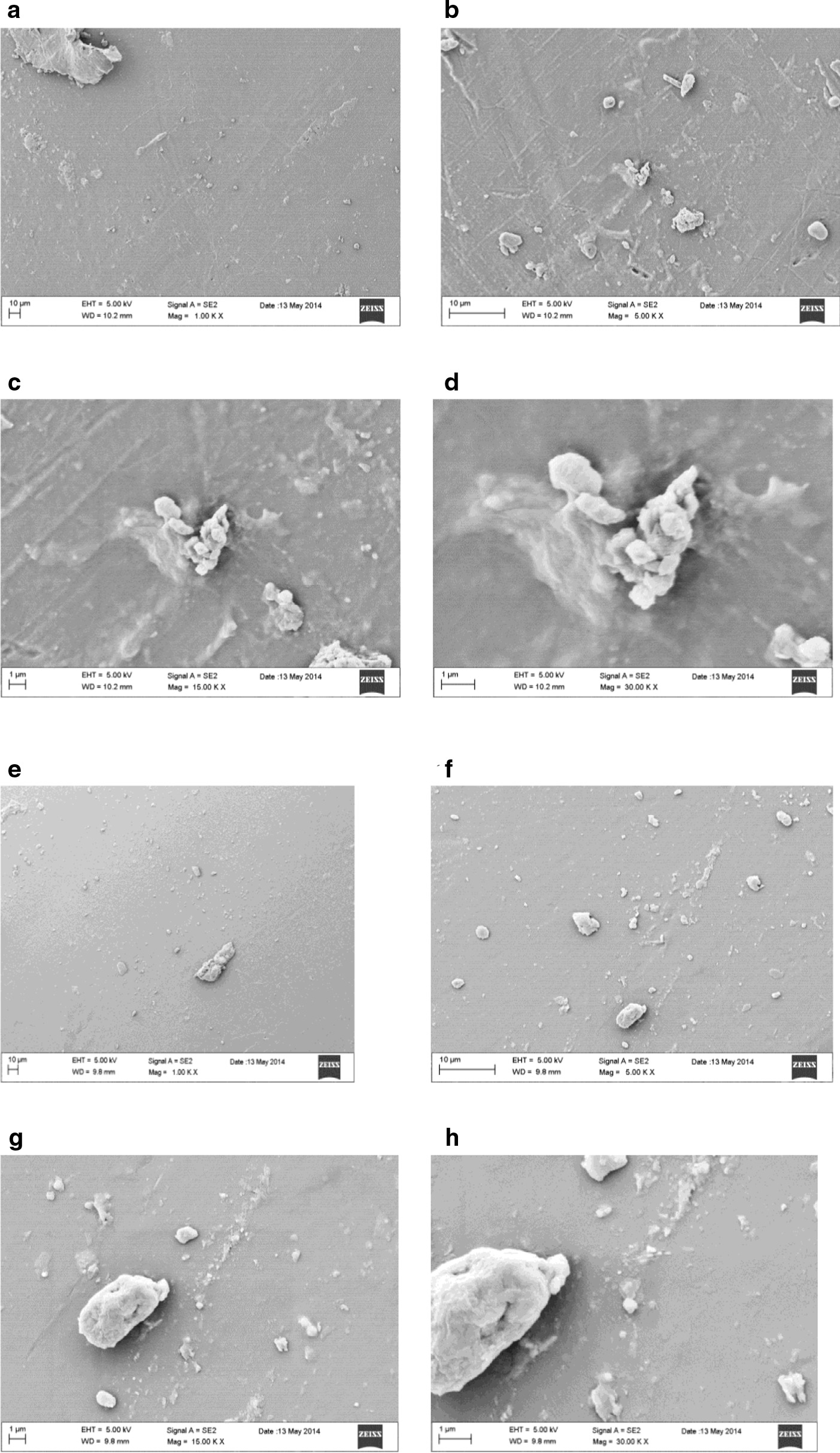
Magnification from 50 to 500X of never used LD30 samples surface

Figure 1 shows the surface details of LD30N samples at magnification from 50 to 500X.

**Fig. 2:**
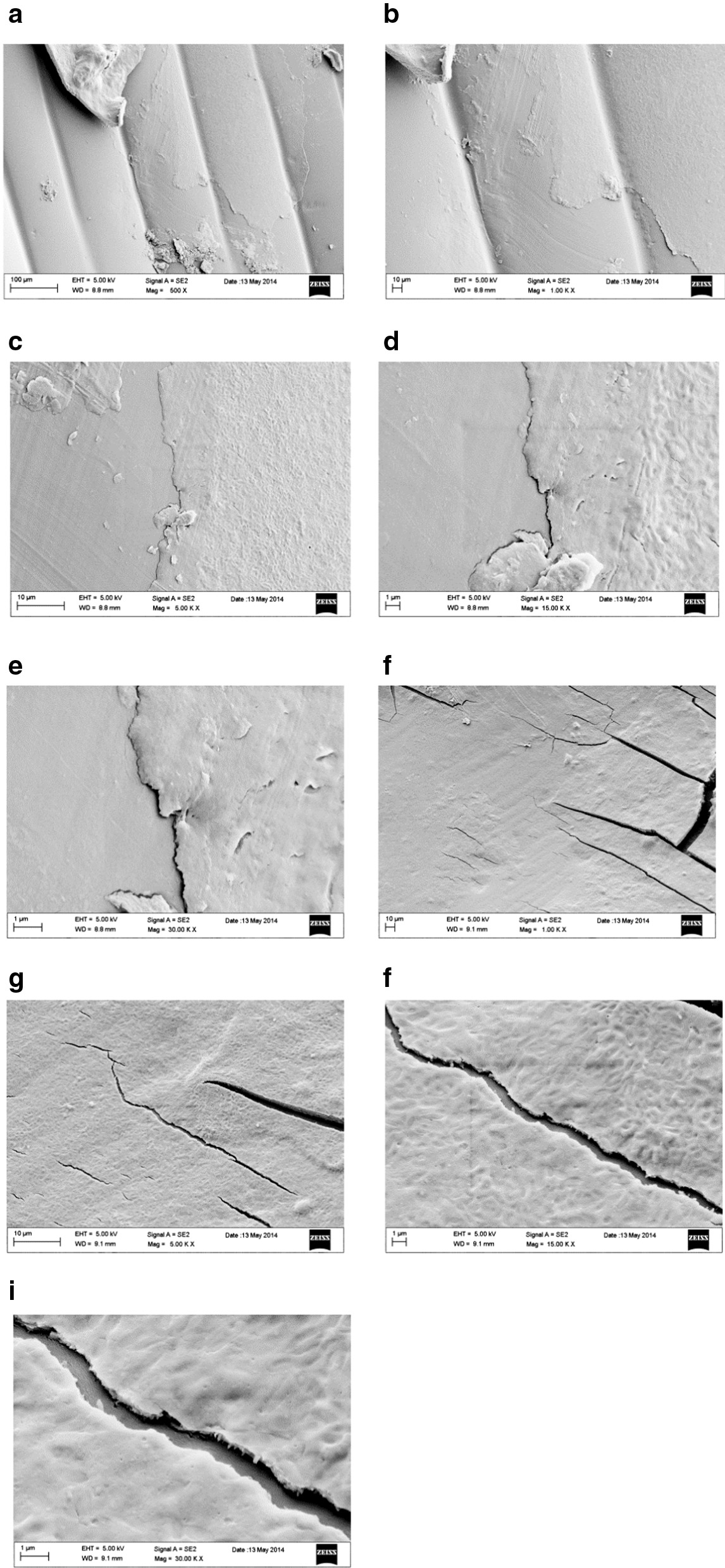
Magnification from 50 to 500X of used LD30 samples surface

In the images of Fig. 2 the superficial characteristics of LD30U at magnification from 50 to 500X are observed.

**Fig. 3:**
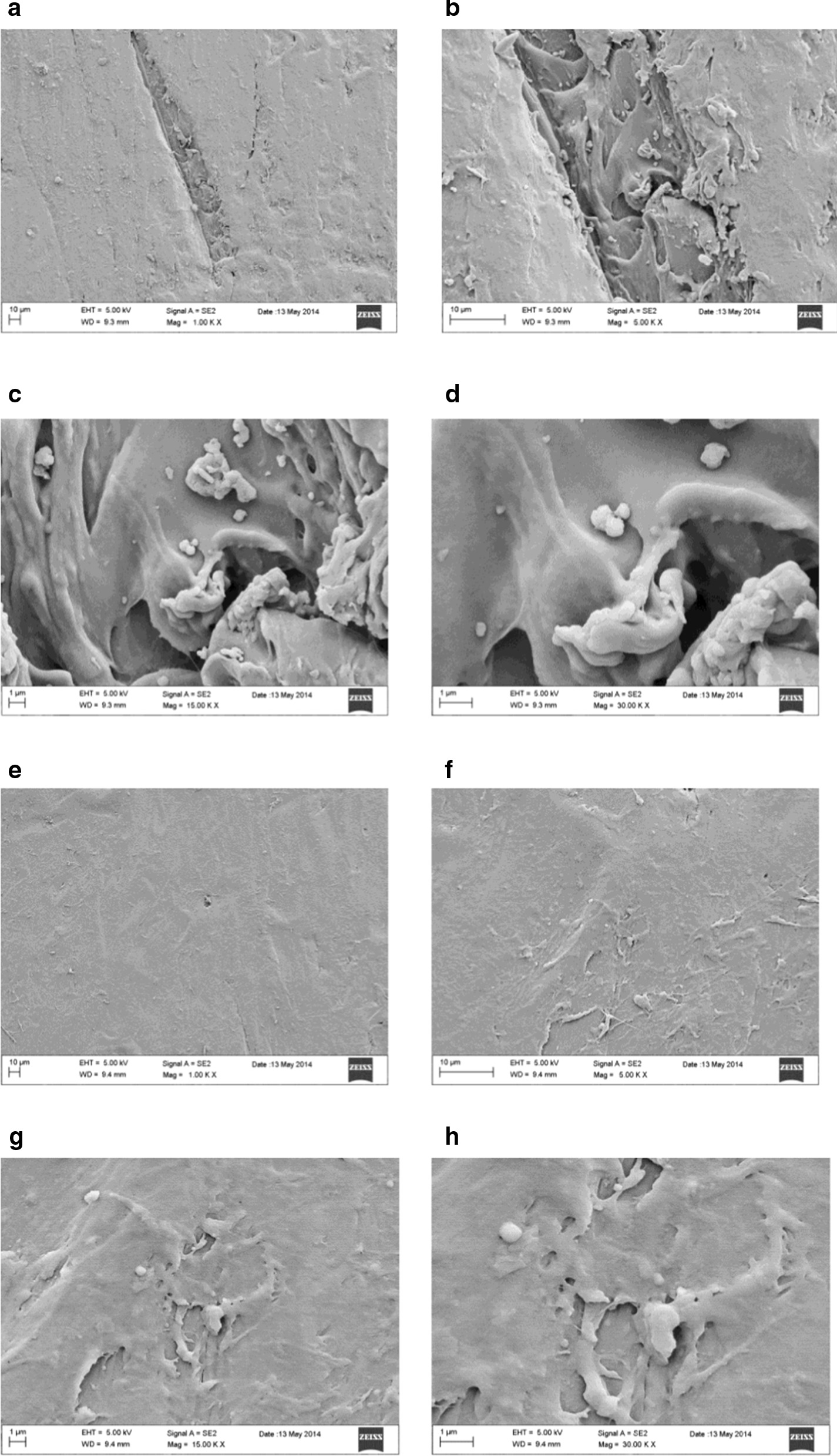
Sequence of magnifications of the never used EX30 samples surface

Figure 3 shows a sequence of magnifications (50X to 500X) of EX30N samples.

**Fig. 4:**
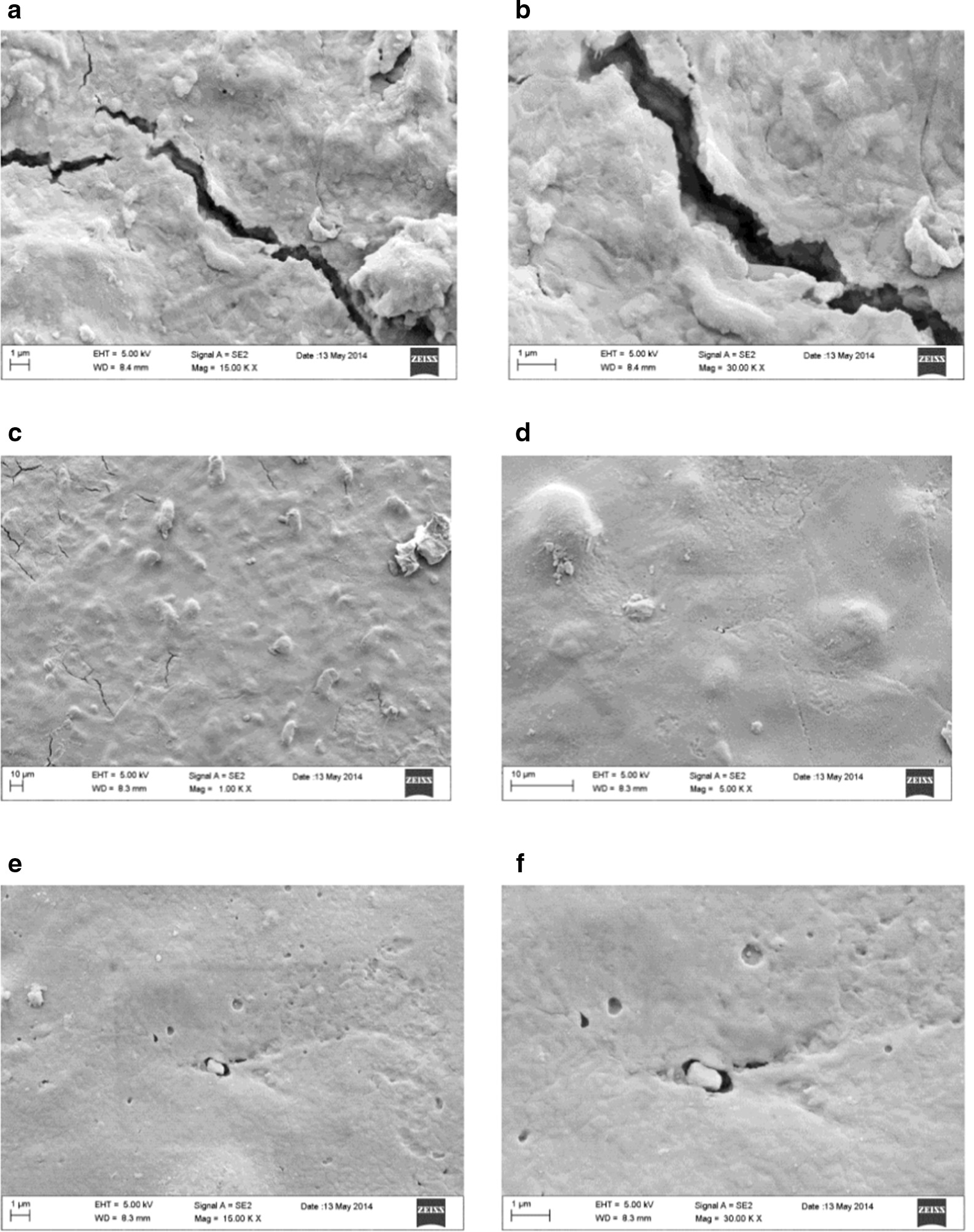
Sequence of magnifications of the used EX30 samples surface

Figure 4 exhibits a sequence of magnifications (from 50 to 500X) for EX30U samples surface.

### Ageing tests results

To investigate real ageing effect on the tested materials, we simulated the aggressive environment present in the mouth with in vitro measurements with PBS and H_3_PO_4_ for increasing times. In case of orthophosphoric acid, although a 10 M concentration of H_3_PO_4_ determines a very aggressive solution, unlikely in a real contest, the purpose was to verify the resistance of the materials respect a critical condition. The use of orthophosphoric acid is justified by the wide use, at lower concentrations, in food industry: indeed, it is often added to drinks to give them a sharped flavor and make drinks more resistant to bacteria proliferation.

**Fig. 5:**
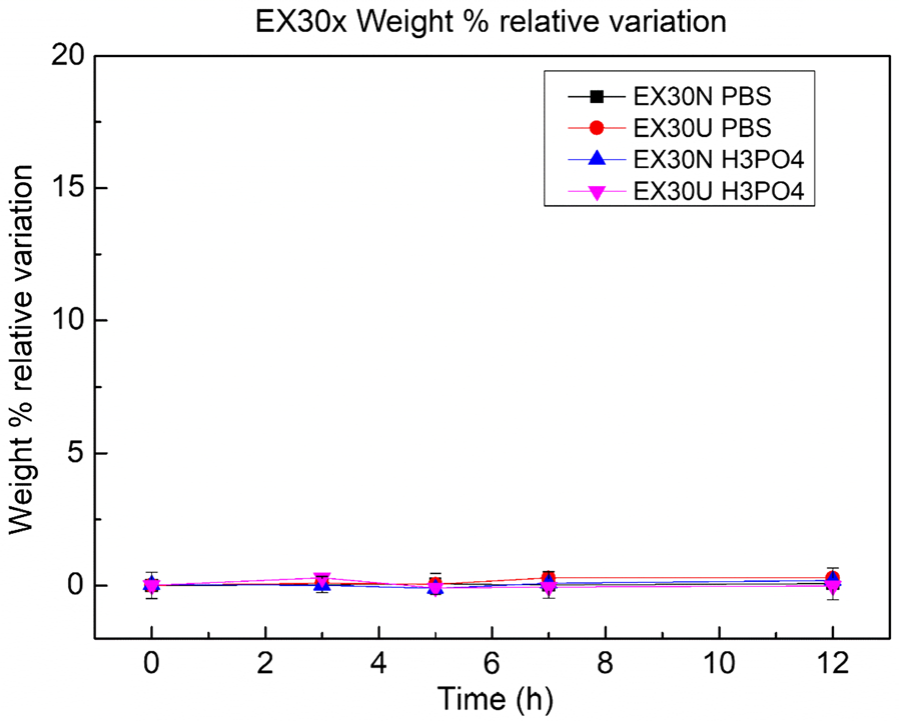
Variations in weight for samples of EX30, never used and used, after their exposure in PBS and H_3_PO_4_ solutions at different time intervals

No one of the samples, after the ageing procedure, releases any compound in terms of weight loss. Conversely, in one case (LD30N), an observable absorption of orthophosphoric acid has been measured. The EX30N samples immersed in H_3_PO_4_ show not significant variations at the beginning of the test. After 3 h of immersion, a small absorption around 2% is detected while, after 5 h of immersion in the acid solution, a lesser reduction in weight can be observed then, for longer immersion, the initial values have been recovered. We can conclude that, EX30 samples weight remains sufficiently stable during all the ageing processes tested (Fig. 5).

**Fig. 6:**
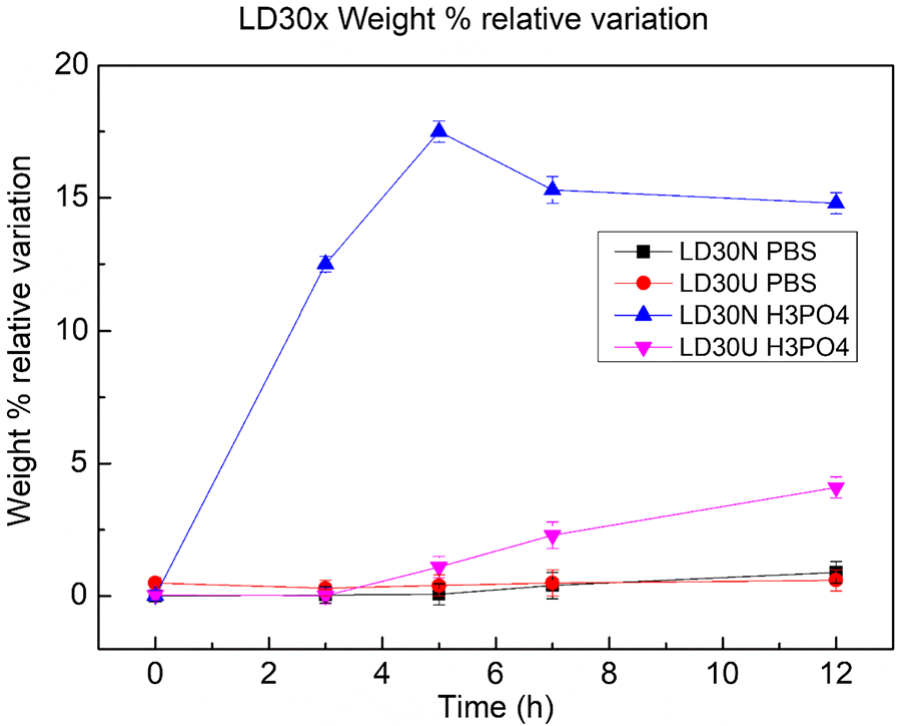
Variations in weight for samples of LD30, never used and used, after their exposure in PBS and H3PO4 solutions at different time intervals

Conversely, LD30N is subjected to a small increase in weight (about 2%) at the first contact with PBS and a higher increment (almost 20%) when subjected to largely acid solution of H3PO4, not appreciable in further uses. The increment in weight observed on the samples of LD30N material indicates that there is an initial absorption of the solution at first use, also if this phenomenon seems to be significant when the material experiments more aggressive conditions (Fig. 6).

## Discussion

SEM analysis, at 50X magnification of LD30N samples, shows that they owning a rather homogeneous and flat surface morphology characterized however by the presence of micrometric detected circular particles, little and sparse superficial alterations and a bigger irregular impurity perhaps remaining from the production techniques. Chromatic variations on the gray scale, at times luminescent and striated in shape probably induced by the modification in the density of the material are observed. In increasing magnification at 100X it is possible to note that the micrometric circular particles and the little alterations resemble indeed rather small conglomerates arranged randomly above a multilayer dense surface which has occasionally even small shallow pits and miscellaneous presence of numerous tiny impurities. Magnifications of 200X and 500X allow observing the complex and rambling form of the agglomerates emerging from the surface plan and become single roundish elaborate structures (Fig. 1).

After the oral usage, the positioning of LD30 aligners on the dental arch is the cause of significant changes in the surface morphology. At 50X magnification of LD30U samples, above the more homogeneous and smooth surface, are noted aligned channels different in depth, superficial cracks and micro/macro-fractures. In particular, the majority of the latter are arranged in parallel according to an oblique axis; only one profound crack is placed perpendicularly to them. Furthermore, with increasing in magnification at 100X and 200X, channels show jagged and uneven edges not very deep, revealing an underlying compact layer. At 500X magnification, the surface layer appears partially covered with a laminar structure jagged, grainy and marked by two large well-defined parallel grooves of equal thickness (Fig. 2). A first clinical consideration arises from the objective assessment that oral use causes the appearance of different alterations over and within the polymer mixture structure. This would indicate that in general the degree of elasticity of the material is not in any case sufficient to counteract the stresses induced by the occlusal load, rather than by the insertion and disengagement maneuvers of the aligners and therefore it is not in any case able to ensure the initial structural integrity. Consequently, reasonable doubts about the ability of the material to transmit orthodontic forces in a constant and homogeneous way arise and with them also the hypothesis that a change, even if minimal, in the modalities and intensity of transmission or propagation of forces within a system closed and programmed a priori seems to be legitimate. It is also possible that the appearance of these alterations can modify the fitting of the aligner making it looser and less adherent to the tooth surface or that in the worst case they can even cause it to break. EX30N samples enlarged at 50X show a higher degree of surface roughness and porosity than LD30 ones. Magnifications of 100X and 200X allow detecting the presence of surface particles, in the sub-micrometric range that appear smaller than those observed for LD30N samples. Further enlargements show an extensive area of shallow depression. At 200X and 500X enlargements, this area is rich in imperfections similar to irregular structures cavernous and laminated to concentric shape that deepen at different levels, with adhering small roundish conglomerates, probably in result of a defective pouring of the polymer during manufacture phases. In addition, changes in the gray scale are much more pronounced in EX30N than in LD30N samples assuming large and significant intervals of chromatic excursion caused by very different depths that seems to indicate the coexistence of differential densities on the same surface (Fig. 3).

In enlargements from 50 to 200X of EX30U, the samples demonstrate a less bumpy and rough surface that the clinical use transforms in a more homogeneous and smooth layer but, even here some fractures are observable, moreover fissures, holes and porosity are also there. Size of fractures in EX30U samples seem to be smaller in micrometric range than those observed in LD30U on the other hand they are much more irregular and unpredictable in arrangement, width and depth (Fig. 4).

Therefore, the structural modification of both materials was not rather similar after use, some considerations arise precisely from the comparison of the two different polymeric mixtures before and after clinical use: in LD30N a much smoother surface is appreciated, even if characterized by the presence of conglomerates of different size and shape but still more homogeneous than that observed on EX30N samples, while after only two weeks in the oral cavity the surface of LD30U becomes more regular due to the disappearance of most of the conglomerates, but at the same time also rougher and characterized by superficial channels and cracks while in EX30U what is striking is the appearance of a greater irregularity and surface porosity in which large cracks are also highlighted. The main differences between LD30 and EX30 are in their original structures, being the latter more amorphous.

In the literature it is reported that the choice of the most suitable base material for the construction of orthodontic thermoplastic appliances strongly depends on the knowledge of the chemical-physical properties typical of each polymeric mixture [17].

Unlike classic orthodontic techniques, the mechanical features of the various polymers are the result of specific manufacturing processes. These properties are also able to qualitatively affect the orthodontic force that the thermoplastic appliance is able to transfer to the dental arch [17].

In this regard, it is known that the effectiveness of the orthodontic movement obtainable through the use of a thermoplastic appliance is lower than that demonstrated by a fixed one [16].

Short-term and long-term load forces act continuously on orthodontic appliances when they are inserted into the oral cavity; the forces of mastication exercised in the night are known as long-term loads, even the insertion or removal of the aligner from the dental arch can be considered loads, but in the short-term [18]. In order for tooth movement to occur, the thermoplastic appliance must continuously be able to transfer controlled orthodontic forces, despite being subjected to continuous and prolonged masticatory loads.

The viscoelastic nature of the thermoplastic material surely represents a disadvantage as the force it is able to generate through the controlled movement tends to decrease over time [17]. Therefore, we look for a thermoplastic material with a constant linear elastic behavior and a high point of return: between the two polymer blends in exam, certainly the *new generation* LD30 better meet these requirements than the previous EX30, but being less amorphous than the previous one, it is certainly more likely to easily break.

Liu et al., comparing at SEM the colour stabilities of three types of never used orthodontic clear aligners exposed to staining agents in vitro, observed that different surface alterations to the three types of aligner materials after the 7-day staining occurred. The three types of aligners exhibited colour stability after the 12-h immersion, with the exception of the Invisalign® aligners stained by coffee. Invisalign® aligners were more prone than the others two brands of aligners to pigmentation. According to this study is aligner materials may be improved by considering aesthetic colour stability properties [19]. Colour is certainly an important physical property for all those dental materials used in order to respect oral aesthetics. A fundamental characteristic of colour is represented by its stability over time and in conditions of use, i.e. within a hostile environment like that represented by the oral cavity. Also in the present study it was possible to observe that both polymeric mixtures under examination underwent appreciable chromatic variations both after 14 days of clinical use than after in vitro immersion in two different test liquids, thus influencing the aesthetic value of the aligner. According to a common chromatic scale EX30U samples tend towards the brown while LD30U ones towards that of the yellow colour.

This suggests that between the two materials under examination, LD30 tends to be more sensitive to liquid substances, since it is weakly more hydrophilic than EX30 and tends to absorb more in the presence of acid solutions that are the cause of the increase of its surface porosity. This change can favour a greater absorption capacity, also if limited to very aggressive cases (10 M H_3_PO_4_).

Presumably the absorption capacity, in addition to an increase in weight, will also be the cause of an expansive volumetric change. From a clinical point of view, this chemical instability produces an important internal structural change that can, for example, affect the retention capacity or adhesion of the aligner to the dental arches: often only a few hours after the first fitting, the aligner results to the patient looser and less tenacious to the dental arch and this cannot derive only from a physical adaptation of the individual. The fitting of aligners on anchorage teeth is a crucial factor in clear aligner orthodontics [20, 21].

The search for a material that undergoes increasingly less significant or in any case permanent structural changes, for example due to repeated mechanical stresses, such as those deriving from the occlusal load and / or as a consequence of the repeated daily manoeuvres of insertion and removal of the orthodontic aligner from the dental arch, as well as a material that is indifferent to the temperature and pH excursions to which the oral cavity is continuously subjected, could represent the ideal situation. At the moment, it is also possible to do a lot from a clinical point of view, as behavioural rules to suggest to patients could certainly be interesting. Of course, avoiding chewing with the aligners worn and avoiding acidic pH drinks can also be a sensible recommendation. Furthermore, carioreceptive subjects, therefore with basically acidic saliva, should be invited to use substances that buffer the salivary pH and also the aligners could be kept for fewer days, not 10 but also 7/8, in the event that the orthodontic treatment allowed, to the aim to limit, as far as possible, the deformation of the aligner.

Finally, it is important to note that since no significant weight loss was recorded in the two mixtures for orthodontic aligners, this must comfort us on the safety of the manufacture technology that allows to avoid the dangerous elution of monomers in the oral cavity, even in critical situations, eluding the current problem of micro-plastics.

## Conclusions

Two generation aligners surface morphology has been in vitro investigated before and after the clinical use and the weight loss of the polymers together with their chemical stability after immersion in different hostile environments has been studied. At SEM, in the used samples, the appearance of surface modifications such as cracks and delamination phenomena, suggests to reduce the time of the single usage to 7/8 days in order to maintain the aligners clinical efficacy for the entire treatment. Accelerated aging tests have shown that both materials have good chemical stability over time and do not lose weight even if they were subjected to high concentrations of acid (10 M). These data are very comforting because, even if to a small extent, they suggest a certain biological safety of both generations of materials.

Although LD30 seems to represent the expression of the technological evolution of EX30, this is made evident above all by its morphological architecture, more homogeneous and defined, in reality it has proved to be more sensitive to liquid substances, since it is weakly more hydrophilic than EX30 and tends to absorb more in the presence of acid solutions that are the cause of the increase of its surface porosity, effectively favouring greater bacterial adhesion.

## Data Availability

The datasets used and/or analyzed during the current study are available from the corresponding author on reasonable request.

## Abbreviations

LD30: Smart Track
LD30N: Smart Track never used
LD30U: Smart Track used
EX30: Exceed 30
EX30N: Exceed 30 never used
EX30U: Exceed 30 used
SEM: Scanning electron microscopy
mm: Millimeter
ml: Milliliter
M: Molarity
°C: Degree Celsius
